# Prediction of New-Onset Diabetes Mellitus within 12 Months after Liver Transplantation—A Machine Learning Approach

**DOI:** 10.3390/jcm12144877

**Published:** 2023-07-24

**Authors:** Sven H. Loosen, Sarah Krieg, Saket Chaudhari, Swati Upadhyaya, Andreas Krieg, Tom Luedde, Karel Kostev, Christoph Roderburg

**Affiliations:** 1 Department of Gastroenterology, Hepatology and Infectious Diseases, University Hospital Duesseldorf, Medical Faculty, Heinrich Heine University Duesseldorf, 40225 Duesseldorf, Germany; sarah.krieg@med.uni-duesseldorf.de (S.K.); tom.luedde@med.uni-duesseldorf.de (T.L.); christoph.roderburg@iqvia.com (C.R.); 2 IQVIA, Bangalore 560103, Karnataka, India; saket.chaudhari@iqvia.com (S.C.); swati.upadhyaya@iqvia.com (S.U.); 3 Department of Surgery (A), University Hospital Duesseldorf, Medical Faculty, Heinrich Heine University Duesseldorf, 40225 Duesseldorf, Germany; andreas.krieg@med.uni-duesseldorf.de; 4 Epidemiology, IQVIA, 60549 Frankfurt, Germany; karel.kostev@iqvia.com

**Keywords:** LT, OLT, diabetes mellitus, immunosuppression, incidence, AI, machine learning

## Abstract

Background: Liver transplantation (LT) is a routine therapeutic approach for patients with acute liver failure, end-stage liver disease and/or early-stage liver cancer. While 5-year survival rates have increased to over 80%, long-term outcomes are critically influenced by extrahepatic sequelae of LT and immunosuppressive therapy, including diabetes mellitus (DM). In this study, we used machine learning (ML) to predict the probability of new-onset DM following LT. Methods: A cohort of 216 LT patients was identified from the Disease Analyzer (DA) database (IQVIA) between 2005 and 2020. Three ML models comprising random forest (RF), logistic regression (LR), and eXtreme Gradient Boosting (XGBoost) were tested as predictors of new-onset DM within 12 months after LT. Results: 18 out of 216 LT patients (8.3%) were diagnosed with DM within 12 months after the index date. The performance of the RF model in predicting the development of DM was the highest (accuracy = 79.5%, AUC 77.5%). It correctly identified 75.0% of the DM patients and 80.0% of the non-DM patients in the testing dataset. In terms of predictive variables, patients’ age, frequency and time of proton pump inhibitor prescription as well as prescriptions of analgesics, immunosuppressants, vitamin D, and two antibiotic drugs (broad spectrum penicillins, fluocinolone) were identified. Conclusions: Pending external validation, our data suggest that ML models can be used to predict the occurrence of new-onset DM following LT. Such tools could help to identify LT patients at risk of unfavorable outcomes and to implement respective clinical strategies of prevention.

## 1. Introduction

Liver transplantation (LT) is a life-saving treatment for patients with end-stage liver disease or acute liver failure [1]. Although the five-year survival rates have risen to above 80%, the long-term sequela of LT are causing significant morbidity and mortality in many patients [2,3]. Most importantly, the use of immunosuppressive medications to prevent rejection of the transplanted liver can lead to several adverse effects, including infections, hypertension, kidney dysfunction, osteoporosis, and malignancy [4,5]. Additionally, patients may experience complications related to the surgery itself, such as bleeding, bile leaks, and thromboses [6,7,8,9]. Long-term complications of LT can significantly impact the patient’s quality of life and increase mortality. However, with appropriate medical management and close monitoring, many of these complications can be prevented or effectively managed [10]. Therefore, regular follow-up care and adherence to medication regimens are crucial for patients who have undergone LT.

Just recently, diabetes mellitus (DM) has emerged as a previously underestimated complication following LT, which can primarily be attributed to the interference of postoperative immunosuppressive therapy with the patient’s glucose metabolism, leading to high blood sugar levels and the development of DM. DM after LT can increase the risk of infection, wound healing complications, and cardiovascular disease, all of which ultimately reduce the patient’s quality of life and survival rate [11,12,13,14]. It is important to acknowledge that the risk of developing DM after LT is dependent on several factors, including the type and dose of immunosuppressive drugs, age, family history, and preexisting medical conditions [15]. Thus, close monitoring of blood glucose levels, lifestyle modifications, and appropriate medication management are essential for the treatment of DM after LT [10]. Nevertheless, predicting which individual patients will develop DM after LT has thus far remained challenging. For this purpose, machine learning (ML), which essentially uses artificial intelligence to build more efficient and effective predictive models than traditional methods by detecting hidden patterns in large data sets, could be used. To date, there are very few studies that have applied ML in LT medicine [16].

Accordingly, the aim of our study was to test the application of innovative ML models to predict the development of new-onset DM after LT (NODALT) and to demonstrate their potential usefulness. If validated, these tools could help identify LT patients at increased risk for adverse outcomes and improve individualized prevention strategies after LT.

## 2. Methods

### 2.1. Datasource

This study used data from the Disease Analyzer (DA) database (IQVIA). Details on the methodology of the database have been published elsewhere [17]. In brief, the DA database contains data pertaining to demographic variables, diagnoses, and prescriptions from general and specialist practices in Germany. Data quality is assessed monthly based on several criteria, including completeness of documentation and linkage between diagnoses and prescriptions. The selection of general and specialist practices for inclusion in the DA database is based on statistics published annually by the Bundesärztekammer (German Medical Association), which provides data on physician age, specialty group, community size category, and federal state. Currently, the DA database includes approximately 3% of all practices in Germany and is representative of these practices [17].

### 2.2. Study Population and Outcome

**Figure 1:**
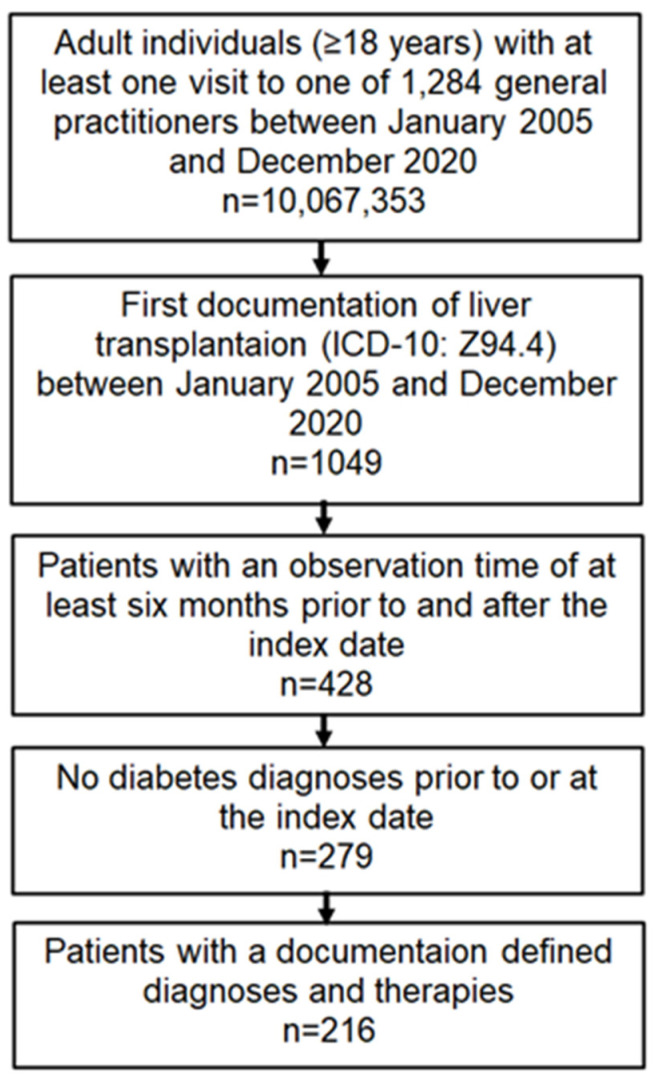
Selection of study patients.

This study included patients aged ≥18 years with a first documented LT (ICD-10: Z94.4) in 206 general practices in Germany between January 2005 and December 2020 (index date). Further inclusion criteria included an observation period of at least six months prior to the index date and a follow-up period of at least six months after the index date. Patients with a diagnosis of DM (ICD-10: E10-E14) before or at the index date were excluded (Figure 1). The outcome of the study was a first diagnosis of DM, including type 2 DM (ICD-10: E11), other specified DM (ICD-10: E13), and unspecified DM (ICD-10: E14), which were documented within 12 months of the LT documentation.

### 2.3. Potential Predictors and Statistical Analyses

The objective of the ML model was to differentiate between patients who were diagnosed with type 2 DM and those who were not, and to identify key predictors of DM diagnosis. The test and training data were randomly split into 80% training and 20% test data and then stratified. According to the reference papers and research conducted, this is a valid method used by many researchers for clinical data. During the split into training and test data, the data were shuffled by means of the random_state parameter in the train_test_split library using the Python programming language. 

Three different ML-based models were run. In the first model, potential predictors corresponded to diagnosis and prescription data obtained within 12 months prior to the index date and within 12 months after the index date. In the second model, potential predictors corresponded only to diagnosis and prescription data obtained within 12 months prior to the index date. In the third model, potential predictors included only diagnosis and prescription data obtained within 12 months after the index date. Since the third model had a much higher area under the curve (AUC) value (0.58) than model 1 (0.45) and model 2 (0.49), we decided to consider the third model for further analyses. The third model included a total of 36 different variables, including age, sex, and the most commonly documented diagnoses (ICD-10 codes) and therapies (ATC codes) (Table 1).

We initially analyzed which diagnoses and therapies were documented in the study population. Over 2300 unique diagnoses and 431 unique therapies were documented for all patients. All diagnoses and therapies present in less than 30% of the population were removed. This method was necessary to measure the impact of the most common diseases and therapies on the outcome as well as to reduce very sparse diagnoses and therapies as the number of inputs to the predictive model. Both diagnoses and therapies were transformed into two types of variables, including frequency of documentation (= frequency) and time between documentation of LT and first documentation of diagnosis or prescription after index date (= time). Systemic corticosteroids were not included as a variable in the model since 100% of study patients received this therapy. Three ML-based methods for predicting DM were tested: random forest (RF), logistic regression (LR), and eXtreme Gradient Boosting (XGBoost).

Since the RF model consists of multiple decision trees, it is helpful to begin with a brief description of the decision tree algorithm. A decision tree is similar to a flowchart tree structure consisting of root nodes, branches, internal nodes, and leaf nodes. It is used to classify input data points or to predict output values for a given input. The input data are processed by asking a series of if-then-else true/false feature questions as well as by estimating the minimum number of questions required to assess the probability of arriving at a correct decision. 

The RF algorithm uses decision tree learning by building a large number of decision trees and then aggregating their output using bootstrap aggregation (bagging). Bagging works by creating multiple subsets of training data that are randomly selected with replacement. At this juncture, each subset of data is used to train the decision trees. Majority voting, i.e., the most frequent categorical variable across the different trees, will yield the predicted class, which in turn will be more robust and have less variance than a single tree. RF is a slightly modified version of bagging where, in addition to taking into account the random subset of data, it also takes into account a random selection of features rather than using all features to grow trees, thus creating an uncorrelated forest of decision trees [18].

Gradient boosting is a method to improve any ML model by iteratively training new models that specialize in addressing the weaknesses of the previous models. Unlike random forest bagging algorithms, which only minimize variance and overfitting of the model, boosting minimizes bias and underfitting. In laymen’s terms, gradient boosting functions by building a weak model from the training data, and subsequently building another weak model that follows the previous model and then attempts to correct its errors. This process continues until either the entire data set has been correctly predicted or the maximum number of models have been added. The technical operation of gradient boosting methods can be explained by two concepts: namely, the gradient descent algorithm and the loss function. The gradient descent algorithm is based on a convex function that can establish a relationship between the actual output and the predicted output. At each point, the derivative (or slope) is found, and we observe the steepness of the slope. This slope will determine the parameters, i.e., the weights and the bias. As the number of successively built weak models increases, the steepness ought to gradually decrease until it reaches the point of convergence. The loss function measures the error between the predicted output and the actual output at the current position. It then provides feedback to the model so that it can adjust the parameters to minimize the error. It continuously iterates along the direction of steepest descent until the cost function is either close to or at zero. At that point, the model stops learning. XGBoost builds trees in parallel, rather than sequentially as is typical in traditional gradient boosting decision trees. It follows a level-wise strategy, scanning across gradient values and using these partial sums to evaluate the quality of splits at each possible split in the training set [19].

LR estimates the probability of an event occurring based on a given set of independent variables. The odds are defined as the probability of success divided by the probability of failure. LR uses a logistic function to transform the odds. The model runs multiple iterations to calculate the log-likelihood function, and LR attempts to maximize this function to find the best parameter estimate. Once the best parameter is found, the conditional probability of each observation is calculated, logged, and summed together to obtain a predicted probability. For binary classification, the probability greater than 0.5 predicts 1 [20].

## 3. Results

### 3.1. Characteristics of the Study Sample and Incidence of DM

The selection of study patients is shown in Figure 1. A total of 216 patients who met all inclusion criteria were included in the study, among which 18 (8.3%) were diagnosed with DM within 12 months of the index date. The mean age of the study patients was 54.1 years (SD: 13.5 years); 56.5% of the patients were male. Patients with a new diagnosis of DM were slightly older (56.3 vs. 53.6 years, *p* = 0.260), and the proportion of men was higher in patients with DM than in those without (64.6 vs. 54.8%, *p* = 0.430). None of these differences were significant.

### 3.2. Performance of the DM Prediction Models

**Figure 2:**
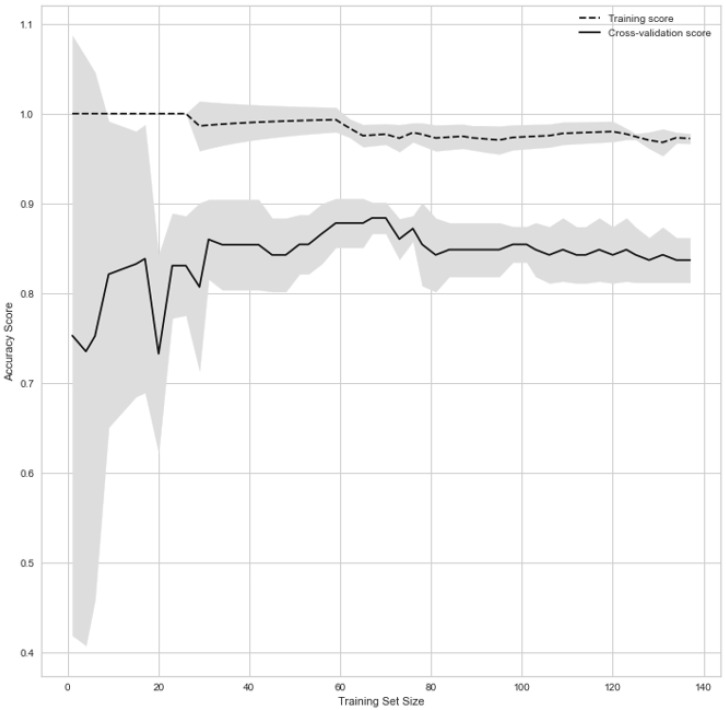
Learning curve.

Since the data are highly unbalanced, we performed a weighted RF, where we assigned a positive weight of 8886110.520507872 and a negative weight of 10 × 10^−4^. The values were derived by running multiple iterations of weight combinations and selecting the most optimal weights. Since the RF classifier tends to be biased toward the majority class, this weight imposes a heavier penalty for misclassifying the minority class. The number of trees in the forest was set to 1000. The Gini impurity scores were used to evaluate the accuracy of the classification. The top features are selected based on their feature importance scores. The learning curve is shown in Figure 2.

**Figure 3:**
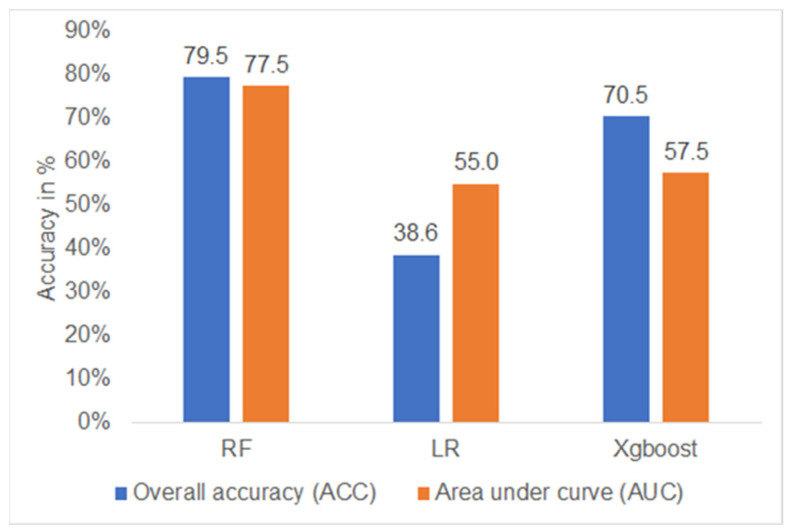
Accuracy of algorithms evaluated. RF—random forest, LR—logistic regression, XGBoost—eXtreme Gradient Boosting.

**Figure 4:**
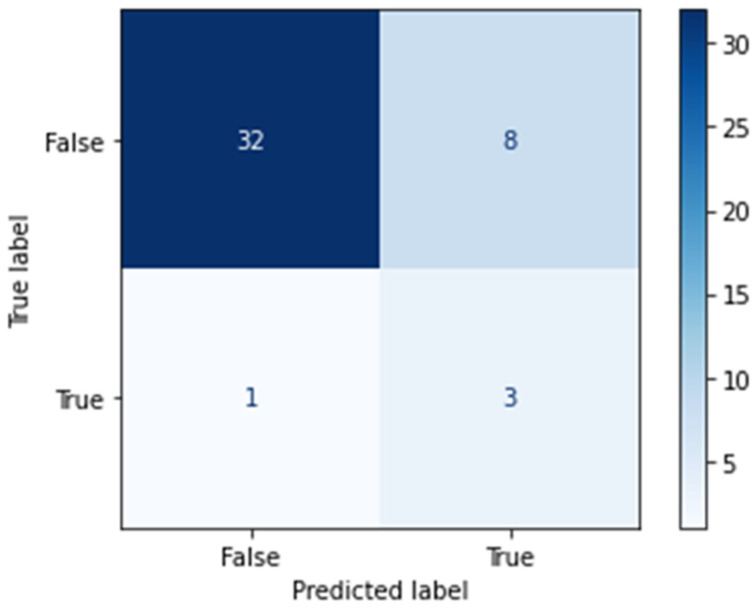
Predicted and true DM patients (random forest).

Figure 3 shows the performance of the three DM prediction models. The performance of the RF model was highest (accuracy = 79.5%, AUC 77.5%). The other two models (LR and XGBoost) showed an overall accuracy and AUC of only 38.6/55.0% (LR) and 70.5/57.5% (XGBoost), respectively (Figure 3). In terms of sensitivity, the RF model was able to correctly identify 75.0% of the DM patients and 80.0% of the non-DM patients in the test dataset (Figure 4).

### 3.3. Important Variables Predicting the Risk of DM

**Figure 5:**
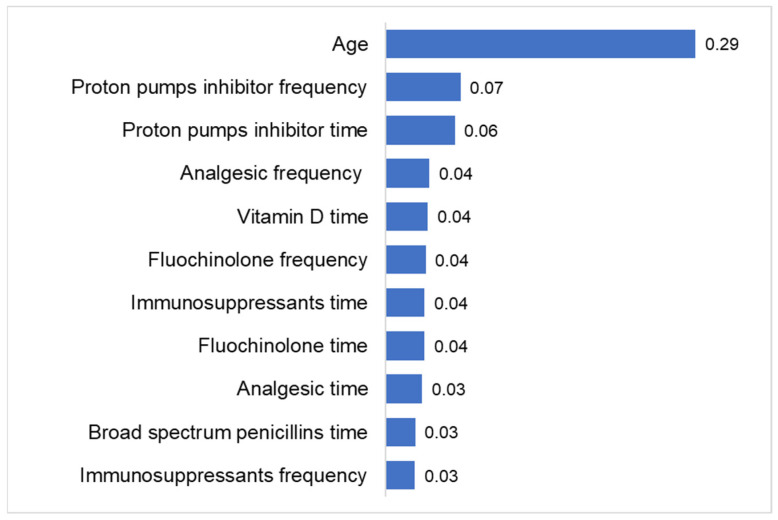
Feature importance in the RF model (top 10 variables + age).

Figure 5 shows the importance of the individual variables on which the RF model was based. The most important feature was age with a value of 0.29 for future importance. The next two features were proton pump inhibitor (PPI) prescriptions with values of 0.07 (frequency of prescription) and 0.06 (time of prescription). Other features of lower importance comprise the prescriptions of analgesics, immunosuppressants, vitamin D, and two antibiotics (broad-spectrum penicillins, fluocinolone; Figure 5).

## 4. Discussion

This retrospective cohort study evaluated three ML models (RF, LR, and XGBoost) on the basis of their predictive performance for NODALT in a German outpatient cohort using the DA database (IQVIA). Remarkably, 8% of patients were newly diagnosed with DM within 12 months of LT. The RF model performed best in predicting NODALT (accuracy = 79.5%, AUC 77.5%), correctly identifying 75.0% of patients with DM and 80.0% of patients without DM in the test dataset. Age was considered to be the most important predictor of NODALT. The PPI prescription followed with values of 0.07 (frequency of prescription) and 0.06 (timing of prescription). Other features of lesser importance included the prescription of analgesics, immunosuppressants, vitamin D, and two antibiotics (broad-spectrum penicillins, fluocinolone).

Although the underlying pathophysiological mechanisms of NODALT remain to be determined, there appear to be unique aspects of this disease [21]. LT is characterized by the graft taking over numerous functions such as digestion, detoxification, and immunity and becomes the patient’s primary metabolic regulator, playing a central role in amino acid synthesis, gluconeogenesis, glycogenolysis, glycogen formation, lipogenesis, and insulin secretion [21]. In this context, the data from Stockmann et al. suggest that the transplanted liver itself may be the origin of metabolic disorders, distinguishing NODALT from new-onset DM after non-liver transplantation [22]. 

To date, there are very few studies that have evaluated ML in LT medicine. These include a review by Spann et al. from 2020 that provides an overview of the strengths and limitations of ML in hepatology and LT medicine [16]. Another study by Bhat et al. analyzed data from adult LT recipients between 1987 and 2016 from the Scientific Registry of Transplant Recipients using different ML methods to identify predictors of NODALT [23]. Consistent with our results, the authors found that increasing age of the recipient was a major contributor to NODALT [23]. In addition, they identified male sex and obesity to be significant recipient factors [23] and, similar to our study, also linked immunosuppressants to the development of NODALT. Against this background, the authors demonstrated that sirolimus as the primary immunosuppressant was associated with a 33% higher risk of post-transplant DM than tacrolimus [23]. However, for donor characteristics not captured in our study, no effect on recipient NODALT risk was observed [23].

Immunosuppressants have previously been associated with the development of NODALT and are thought to have an impact on the deterioration of β-cell function [21]. In addition to systemic corticosteroids—which were not included as a feature in our models since 100% of study patients received this therapy—calcineurin inhibitors, considered as standard immunosuppressants and used for an extended period of time after transplantation, are believed to play a role in the development of NODALT [21]. In this context, experimental studies have shown that tacrolimus negatively affects insulin secretion from β-cells through multiple pathways, including mitochondrial biogenesis, ATP metabolism, membrane trafficking, and cytoskeletal remodeling [21,24,25]. An animal study in obese and lean diabetic rats further demonstrated that preexisting insulin resistance potentiates the diabetogenic effects of tacrolimus through potent inhibition of the Ins2 gene and β-cell proliferation [26]. 

The knowledge of the side effects of immunosuppressive drugs and the immunology of LT has greatly increased over the past years [21]. Due to hepatic immune tolerance, immunosuppressive therapy with low-dose and low-concentration tacrolimus has become a routine protocol in LT patients. Furthermore, mTOR inhibitor combination therapy may allow for a reduction in tacrolimus dose, potentially minimizing tacrolimus-related side effects. However, in clinical trials, no significant reduction in the incidence of NODALT was observed in LT patients treated with tacrolimus minimization therapy (tacrolimus in combination with mTOR inhibitors) as opposed to standard dose therapy [27]. Similarly, another experimental study discovered that even short-term and extremely low concentrations of tacrolimus impaired β-cell secretion by causing a low rate of insulin gene transcription in rats [28]. In addition, corticosteroid-free immunosuppression in LT may be recommended in the future upon discovery of its association with a lower risk of NODALT in a recent evidence-based review [29]. 

Recent research interest has also focused on the gut microbiome, the alteration of which has been discussed in the context of the pathogenesis of glucose metabolism disorder [21,30]. To this end, there is evidence that antibiotics, PPIs, and non-steroidal anti-inflammatory drugs identified in our model as predictive features of NODALT have an impact on the gut microbiome [31,32,33]. A Chinese metagenome-wide association study has found that patients with type 2 DM are characterized by a dysbiosis of the gut microbiome, with a decrease in the abundance of several universal butyrate-producing bacteria and an increase in several opportunistic pathogens, as well as an enrichment of other microbial functions mediating sulfate reduction and resistance to oxidative stress [34]. Interestingly, specific gut microbiota are also found in LT patients [35]. In a pilot study from China that examined six gut bacteria in fresh stool samples, LT recipients showed a significant decrease in butyrate-producing bacteria (e.g., Faecalibacteriumprausnitzii) and an increase in opportunistic pathogens (e.g., Enterococcus spp.) compared to healthy subjects [35], which were similar to the characteristics of patients with DM [34,36]. 

Inflammatory processes and the involvement of lipopolysaccharides (LPS), the major component of the outer membrane of Gram-negative bacteria, which in turn activate toll-like receptors (TLRs) by binding to the LPS-binding protein (LBP), may also play a role in the development of NODALT [37]. It is interesting to note that animal studies in this context suggest a possible impairment of glucose homeostasis in LT recipients by establishing that LT leads to a translocation of LPS in hepatocytes and an upregulation of LBP, resulting in an enhanced inflammatory response [38]. Imbalance of gut microbiota and an impaired gut barrier in liver transplant recipients could ultimately lead to various liver diseases such as inflammation and steatosis as well as to metabolic syndrome via the “gut–liver axis” through various biological mechanisms [37]. 

Correspondingly, the proliferation of proteobacteria is considered to be a hallmark of abnormal microbiota as a result of dietary changes, inflammation, or antibiotic therapy [39]. The finding in our study, which demonstrated that only broad-spectrum penicillins and fluocinolones were identified as predictive features of NODALT in our ML model, may suggest that even single antibiotic drugs could lead to a relevant change in the composition of the gut microbiome. Within this framework, it has been observed that various antibiotics that target the gut have different effects on the density and diversity of the microbiota. Accordingly, a broader spectrum of activity of penicillin and fluocinolone could explain their influence in the development of NODALT via a stronger effect in reducing microbial diversity [40]. However, to better understand the relationship between NODALT and the gut microbiome as well as the effects of antibiotic therapy, further studies focusing on this question are required.

Several studies have indeed observed a change in the composition of the microbiome with PPI use [32,41]. PPIs are among the most commonly used medications worldwide and are primarily used to treat reflux disease and gastric and duodenal ulcers. Long-term use of PPIs has been associated with a number of complications, including kidney disease, bone fractures, intestinal inflammation, and more recently, the development of DM. Yuan et al. analyzed whether regular use of PPIs increases the risk of type 2 DM in three large epidemiologic studies with a total of 204,689 participants who did not have DM at baseline [42] and found a 24% increased risk of DM in individuals who regularly used PPIs. The risk was dependent on the duration of PPI therapy and was lower in individuals who had taken the medication for up to 2 years than in those with a longer duration of therapy [43]. However, to our knowledge, our study was the first to evaluate NODALT in combination with PPIs.

In terms of age, which was identified as the most important feature of NODALT in our study comprising a retrospective analysis of data from 188 adult primary liver transplant recipients in Japan, Abe et al. also found, in agreement with our findings, that older recipient age (≥55 years) was a significant risk factor for the development of DM after transplantation [43]. Similarly, Cosio et al. examined data from 2078 kidney transplant recipients using a multivariate Cox model and found that patients older than 45 years at the time of transplantation had more than twice as high a risk of developing DM after transplant compared with younger patients [44]. In addition, other studies show that the risk of developing post-transplant DM after solid organ transplantation more than doubles with each 10-year increase in recipient age [45]. This effect is claimed to be attributable to the fact that islet cells age and undergo apoptosis with age. Decreased insulin secretion and increased insulin resistance have also been suggested to this end [46].

At this juncture we should refer to some of the limitations of this study. First of all, the sample size is small, especially for the test group. Therefore, further studies with larger samples are required to externally validate the application of the models. Second, the retrospective and observational nature of our study may lead to unavoidable selection bias. The ML algorithms generated are only as good as the quality of the data provided. Thus, several factors may influence the performance and accuracy of an ML model. Since the diagnoses are based on the documentation of ICD-10 and ATC codes by primary care physicians, it cannot be excluded that some of the diagnoses were misclassified. Another limitation of the study is the lack of information regarding the underlying diseases of LT recipients and donors. Furthermore, certain information that would have allowed for additional analyses, such as socioeconomic status, family history, ethnic background/race, environmental conditions, lifestyle factors (e.g., physical activity, nicotine or alcohol use, diet), including etiology, surgery duration, or other factors associated with increased DM risk, such as presence of acute rejection and the number of rejection episodes, dose of glucocorticoid treatment, and family history of diabetes mellitus, were not available. In addition, there was no information available on the genotype of the patients that would have allowed further analyses, especially since the literature has acknowledged that the graft genotype may also influence the metabolic state after LT. In particular, the graft gene variant of the DM susceptibility gene TCF7L2 rs290487 (C allele) has been associated with an increased risk of NODALT [31]. Finally, it is important to consider that this retrospective analysis indicates an association, as opposed to a cause-and-effect relationship.

Pending external validation, our data suggest that ML models may be useful in predicting the occurrence of NODALT. Such tools could help to identify LT patients at risk for adverse outcomes and implement appropriate clinical prevention strategies. Nevertheless, further studies are required to confirm which factors contribute most to facilitating individualized clinical care during post-transplant management.

## Figures and Tables

**Table 1:** Different features.

	ICD-10 or ATC Code	Features	Patients (N, %)	Median Time between Documentation of LT and First Documentation of Diagnosis or Prescription after Index Date (Days).
ATC features (based on the Anatomical Classification of Pharmaceutical Products by the European Pharmaceutical Market Research Association (EPHMRA)	A02B2	Proton pump inhibitors	(25, 11.6%)	85
	A05A2	Bilestone therapy	(18, 8.3%)	51
	A11C2	Vitamin D, plain	(13, 6.0%)	134
	C03A2	Loop diuretics, plain	(11, 5.1%)	62
	C07A0	Beta-blocking agents, plain	(15, 6.9%)	98
	J01C1	Oral broad-spectrum penicillins	(18, 8.3%)	219
	J01G1	Oral fluoroquinolones	(15, 6.9%)	257
	L04X0	Other immunsuppressants	(25, 11.6%)	42
	M01A1	Non-steroidal antirheumatic drugs	(9, 4.2%)	109
	N02B1	Other analgesics	(17, 7.9%)	116
	X25A0	Physical therapy	(13, 6.0%)	117
ICD-10 features	A09.9	Gastroenteritis and colitis of unspecified origin	(4, 1.9%)	261
	I10.0	Essential (primary) hypertension	(15, 6.9%)	83
	J06.9	Acute upper respiratory infection, unspecified	(17, 7.9%)	224
	J20.9	Acute bronchitis, unspecified	(9, 4.2%)	187
	K74.6	Other and unspecified cirrhosis of liver	(4, 1.9%)	86
	M54.1	Radiculopathy	(10, 4.6%)	78
	Z25.1	Need for immunization against influenza	(12, 5.6%)	208
Age			mean = 62.1 years, SD = 14.7 years	
Sex (Female)			(96, 44.4%)	

## Data Availability

The datasets used and/or analyzed during the current study are available from the corresponding author upon reasonable request.
